# Supplementary material for: Brownmillerites CaFeO2.5 and SrFeO2.5 as Catalyst Support for CO Oxidation
Source: Molecules. 2021 Oct 23;26(21):6413. doi: 10.3390/molecules26216413 (PMC8587075; doi:10.3390/molecules26216413)
Supplement: Supplementary file 1 [file molecules-26-06413-s001.zip › molecules-1409294-supplementary.pdf]

Supporting Information

For

Brownmillerites $CaFeO_{2.5}$ and $SrFeO_{2.5}$ as catalyst support for CO oxidation

Pierre-Alexis Répécaud [1,2], Monica Ceretti[2], Mimoun Aouine[3], Céline Delwaulle[4], Emmanuel Nonnet[4]
Werner Paulus[2]* and Helena Kaper[1*]

1 Laboratoire de Synthèse et Fonctionnalisation des Céramiques, CNRS/Saint-Gobain CREE, Saint-Gobain Research Provence, 550, Ave Alphonse Jauffret, 84306 Cavaillon, France;
2 ICGM, University Montpellier, CNRS, ENSCM-34095, Montpellier, France;
3 Université de Lyon, Université Claude Bernard Lyon, CNRS, IRCELYON, 2 av Albert Einstein, 69626 Villeurbanne, France;
4 Competency Research Laboratory, Saint-Gobain Research Provence, 550, Ave Alphonse Jauffret, 84306 Cavaillon, France;

* Correspondence: H.K.: helena.kaper@saint-gobain.com; W.P.: werner.paulus@univ-montp2.fr

Table S1. Physicochemical properties of CFO_cp, CFO_eaf, SFO_cp and SFO_eaf.

	**CFO_cp**	**CFO_eaf**	**SFO_cp**	**SFO_eaf**
Specific surface area (m^2/g)	12	<1	13	<1
Ca:Fe ratio	0.95	1.06		
Sr:Fe ratio			0.99	1.05

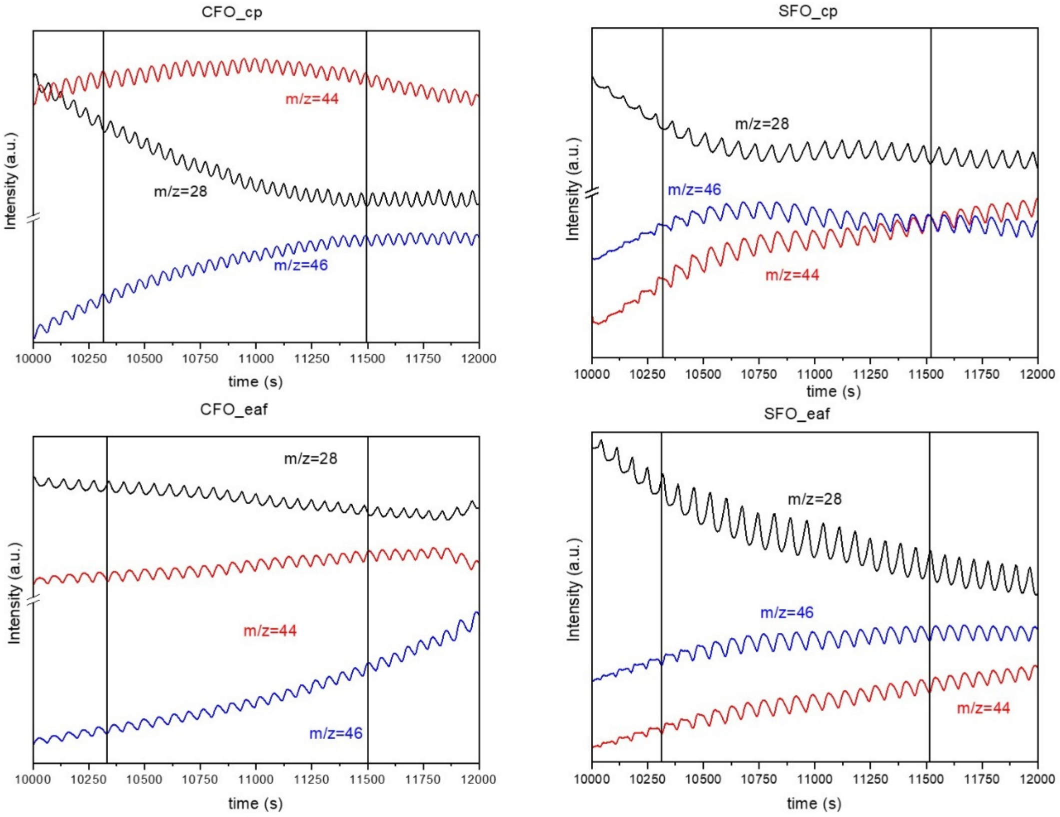

Figure S1. Comparison of oscillation during CO-oxidation on ^{18}O-exchanged materials. Upper left: CFO_cp, upper right: SFO_cp, lower left: CFO_eaf, lower right: SFO_eaf.

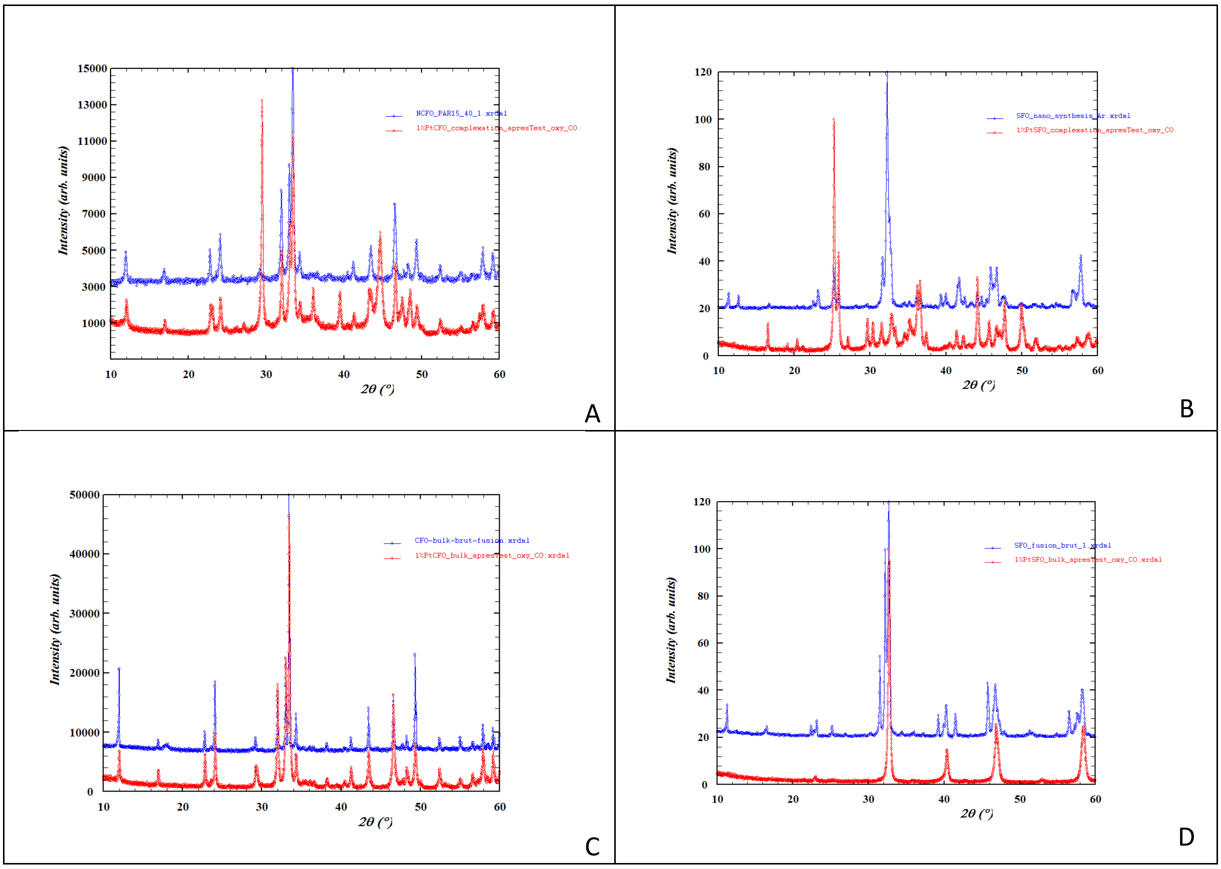
Intensity (arb. units)
2θ (°)
NCFO_PAR15_40_1.xrdml
1%PtCFO_complexation_apresTest_oxy_CO.
A
SFO_nano_synthesis_Ar.xrdml
1%PtSFO_complexation_apresTest_oxy_CO.
B
CFO-bulk-brut-fusion.xrdml
1%PtCFO_bulk_apresTest_oxy_CO.xrdml
C
SFO_fusion_brut_1.xrdml
1%PtSFO_bulk_apresTest_oxy_CO.xrdml
D
